# Therapy Allergen Ordinance (TAO): The final stretch 

**DOI:** 10.5414/ALX02594E

**Published:** 2025-11-14

**Authors:** Vera Mahler, Diana Hartenstein, Iris Lauer, Stefan Vieths, Claudia Ruoff, Julia Zimmer, Susanne Kaul

**Affiliations:** 1 Allergology Division,; 2 President of the Paul-Ehrlich-Institut; Molecular Allergology Research Group, and; 3 Legal Affairs Section, Safety of Biomedicines and Diagnostics Division, Paul-Ehrlich-Institut, Langen, Germany

**Keywords:** named patient products, marketing authorization, efficacy, safety, quality, benefit-risk ratio, pediatric investigation plan, clinical trial, marketing authorization application

## Abstract

The Therapy Allergen Ordinance (TAO) aims to migrate allergen immunotherapy (AIT) products for the treatment of common allergies, which were previously marketed in Germany as named patient products (NPPs), into authorized products provided their quality, efficacy, and safety are adequately shown. The TAO applies to all NPPs containing active ingredients based on the following allergen sources: house dust mites, bee venom, wasp venom, pollen from sweet grasses (excluding maize), birch, alder, or hazel. The last product-specific deadlines granted under the TAO for the submission of clinical data relevant to the marketing authorization process will expire in 2026. The subsequent final assessment of the updated marketing authorization application (MAA) is carried out by the competent authority, the Paul-Ehrlich-Institut. During this period of processing by the competent authority, the products remain marketable until a decision regarding the MAA has been reached. Currently (as of August 08, 2025), 40 AIT products are still marketable under the transitional provisions of the TAO (10 preparations for the treatment of allergies to house dust mites, 10 against tree pollen allergies, 16 against grass pollen allergies, and 4 mixed preparations containing non-homologous allergen groups). For 8 of these products, pharmaceutical companies have initiated the withdrawal of MAA as of October 01, 2025 or January 31, 2026, respectively. Prior to the final assessment of the updated MAAs, the competent authority is unable to make any public statements as to whether the individual applications for the remaining 32 products under the transitional provision will be concluded positively with the granting of the marketing authorization or its rejection. With the rejection of a marketing authorization, the product-specific marketability ends immediately, i.e., there is no additional sell-off period for the TAO products concerned. If a marketing authorization is granted, the marketability of the specific product is perpetuated.

## Introduction 

The Therapy Allergen Ordinance (TAO), which came into force in Germany in 2008, aims to transfer allergen immunotherapy (AIT) products containing active ingredients from house dust mites, bee venom, wasp venom, pollen from sweet grasses (excluding maize), birch, alder, or hazel and were previously distributed as named patient products (NPPs), into tested and authorized products, provided their quality, efficacy, and safety have been adequately demonstrated. 

The Paul-Ehrlich-Institut (PEI) reports regularly at allergy conferences and in allergology journals on the status of marketing authorization applications for therapy allergens under the transitional provision of the TAO [1, 2, 3, 4]. 

An up-to-date overview of AIT products authorized in Germany as well as of marketable therapy allergens in the marketing authorization process under the transitional provision of the TAO can be found on the PEI website (https://www.pei.de/EN/medicinal-products/allergens/allergens-node.html). 

The TAO is a legal ordinance in which no expiry or termination due to the passing of time is specified. However, the last product-specific deadlines for the submission of clinical data relevant to marketing authorization, as granted by the competent authority in accordance with the transitional provision of Section 3 (4) TAO, will expire in the course of 2026. The PEI is taking this opportunity to report on practical aspects of this phase of the marketing authorization processes under the TAO in this article. 

## Product development under the TAO: Procedural aspects at the start of implementation 

**Figure 1.**
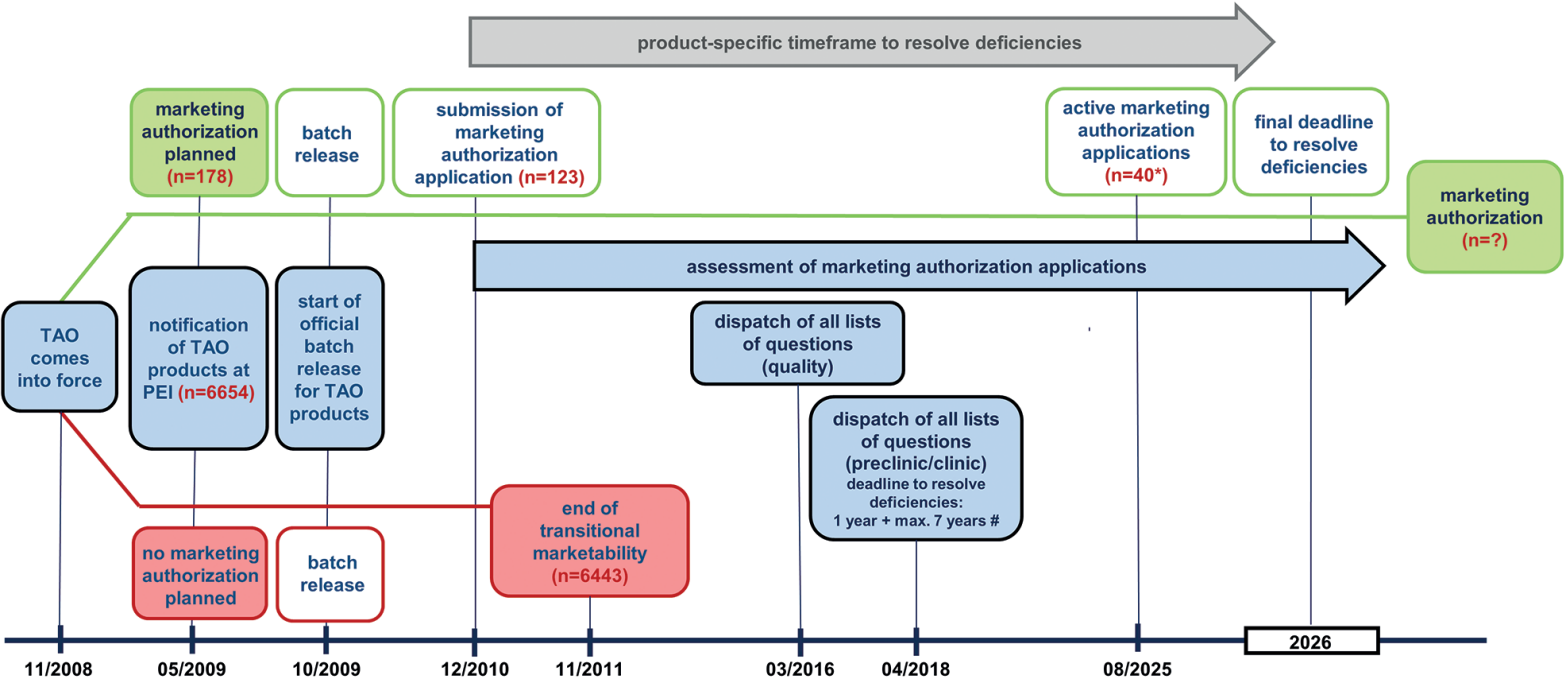
Timeline Therapy Allergen Ordinance (TAO). *At the specified date (August 8, 2025), 40 applications for marketing authorization under the TAO are still active; however, for 8 of these products, withdrawals of the marketing authorization application have already been declared for different future dates of effect. ^#^The maximum period for resolving deficiencies of 1+7 years was allocated fractionally on a product-specific basis, based on the respective status and progress in clinical development.

All NPP of therapy allergen products containing active ingredients from one or more of the above-mentioned allergen sources had to be reported to the competent authority by May 14, 2009 (Figure 1). Five months after reporting, they were subjected to the official state batch testing. 

If marketing authorization was intended, the corresponding application had to be submitted to the PEI by November 30, 2010, which was done for 123 of the above-mentioned NPPs (Figure 1). The European Regulation on Medicinal Products for Paediatric Use [5] ensures that the specific therapeutic needs of children and the particular vulnerability of this population group are taken into account in the development of medicinal products. One requirement of this regulation is that, in general, a pediatric investigation plan (PIP) approved by the Paediatric Committee (PDCO) must be submitted with the marketing authorization application (MAA) of medicinal products. These specifications were followed when the TAO was implemented [3, 6]. To enhance efficiency of the TAO process, a standard PIP for allergen products for AIT was developed at the PDCO level [7]. 

The marketability of the 6,443 NPPs (declared under Section 3 (2) TAO) containing the above-mentioned active ingredients, for which no marketing authorization was sought, ended on November 14, 2011, with the end of the transition period for their placing on the market (Figure 1). 

The transitional provision of the TAO stipulates that for the above-mentioned NPPs for which an MAA has been submitted, the 1-year deadline, starting from the date of receipt of the deficiency letter, may be extended by a maximum of 7 years for submitting clinical data, if this is necessary to remedy deficient clinical data due to the nature of therapy allergens. The last of these product-specific deadlines will expire in the course of 2026 (Figure 1). 

## Study activities under the provision of TAO 

There was a high level of study activity under the provisions of the TAO. A total of 62 applications for clinical trials with NPPs subject to the TAO were submitted to PEI. Of these, 49 were approved (8 of these with conditions). One trial that had already been approved was withdrawn by the applicant. Nine trials were rejected, and 4 others had been withdrawn before the assessment was completed. 41 trials have been completed to date (including 21 dose-finding studies, 11 phase III studies, and 3 combined phase II/III studies). 35 clinical trial reports have been submitted to PEI to date. Where results from dose-finding trials are available, the market dose showed a positive benefit-risk ratio in most cases, but was not always the “optimal” dose: in 7 cases, a higher dose showed a better benefit-risk ratio, and in 13 cases, the result was inconclusive with regard to a dose with superior efficacy. 

If the study results submitted did not demonstrate sufficient efficacy and safety, the competent authority would no longer grant batch release during the ongoing TAO procedure before the expiry of the deadlines granted for marketable TAO products. This means that although the products in question could still be developed under the TAO, no new batches would be allowed to enter the market. This applied to several AIT products [8]. 

## Product development under TAO: Procedural aspects of the final phase 

**Figure 2.**
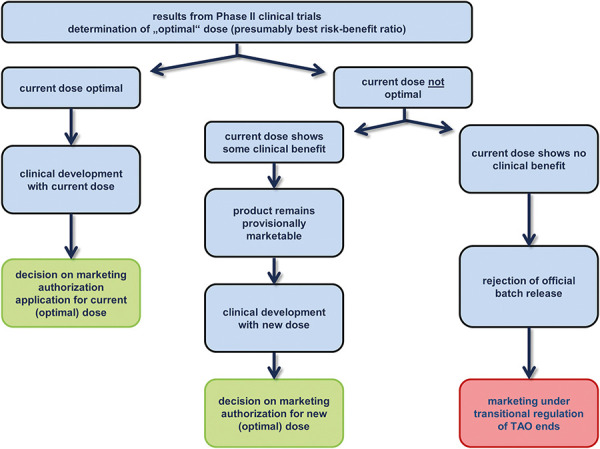
Schematic representation of the regulatory process based on the results of dose-finding studies.

As part of the marketing authorization process, the pharmaceutical company must provide evidence of the sound quality, efficacy, and safety of the AIT product. Marketing authorization can only be granted for products with a positive benefit-risk ratio. Before the product-specific deadline expires, the applicant must therefore submit study data to remedy any existing deficiencies. If the dose-finding studies have confirmed the originally submitted dose, the existing marketing authorization dossier must be updated with the further study data from the pivotal clinical trial(s) if a marketing authorization is still sought for this dose (Figure 2). 

**Figure 3.**
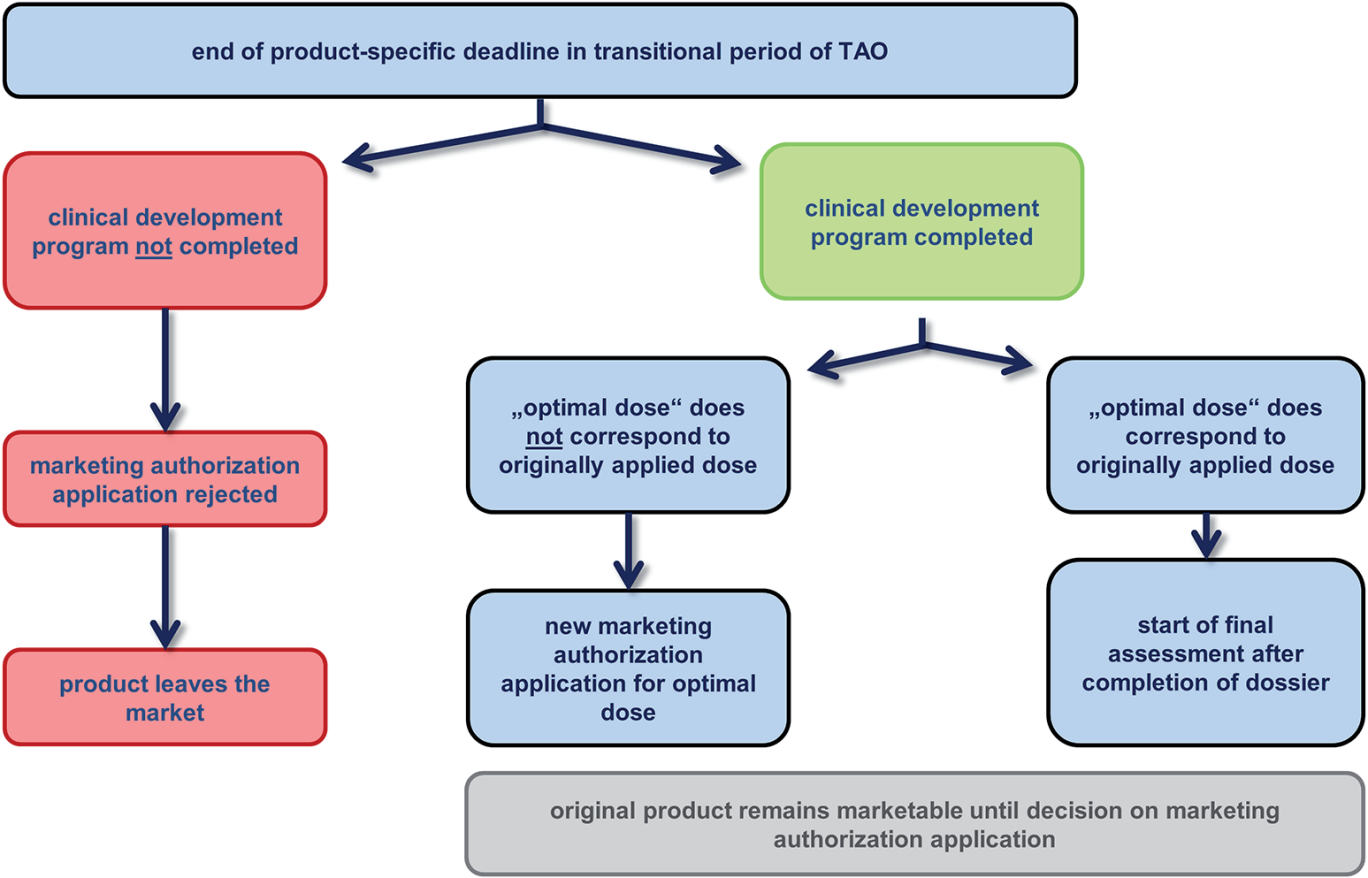
Schematic representation of the regulatory processes at the end of the product-specific period for resolving deficiencies.

However, if the optimal dose differs from the dose of the original MAA and which is marketed under the TAO, a new MAA must be submitted for this modified product in accordance with Sections 21 ff. of the German Medicinal Products Act (Arzneimittelgesetz (AMG)) (Figure 3). The PEI will assess the updated MAA and the new application. The processing times of the competent authority are in addition to the above-mentioned deadlines. The quality of the product is assessed, among other things, in accordance with the European Pharmacopoeia and the “Guideline on allergen products: production and quality issues” (EMEA/CHMP/BWP/304831/2007) [9], and the clinical development plan must comply with the requirements of the “Guideline on the clinical development of products for specific immunotherapy for the treatment of allergic diseases” (CHMP/EWP/18504/2006) [10]. This includes positive results for the investigational drug from phase II and III clinical trials [11]. Phase III (or phase II/III) clinical trials must not only show a statistically significant positive result compared to placebo in the primary endpoint, but also demonstrate the clinical relevance of the treatment. To this end, the study protocol must clearly define how large the difference between the effect of the investigational drug and that of the placebo must be in order to translate into a clinically relevant improvement for patients [3]. In individual cases, the overall assessment of primary and secondary endpoints may also lead to evidence of clinical relevance [3]. If an MAA is based on a single pivotal study, this study must be exceptionally compelling in terms of internal and external validity, clinical relevance, statistical significance, data quality, and internal consistency [12]. 

The final assessment also includes a review of the conformity of the development program with the approved PIP (PIP compliance), which is also a prerequisite for granting a marketing authorization for use of the medicinal product in adults. [3, 13]. 

The TAO procedure ends with a decision to reject or grant the marketing authorization, unless the applicant has withdrawn the MAA before that. The assessment is based on the updated dossier. The marketability of the medicinal product ends when the rejection decision is announced or when the withdrawal of the application takes effect (Figure 3). In case of granting marketing authorization, follow-up procedures are governed by the relevant provisions of the German Medicinal Products Act. 

If the final assessment of the marketing authorization dossier shows that all deficiencies in terms of quality and clinical aspects have been solved and PIP compliance has been confirmed [13], the marketing authorization is granted for the product marketable under the TAO or for the optimized AIT product developed on the basis of the TAO product (Figure 3). In the latter case, the marketability of the originally marketed product ends with the marketing authorization of the optimized product. 

If the clinical deficiencies of an AIT product have not been solved by the final deadline granted under the TAO, the PEI will reject the marketing authorization. The AIT product, which was marketable under the transitional provisions of the TAO, may then no longer be placed on the market from the date of rejection, regardless of whether an updated dossier for the marketed product or a new application for an optimized product (e.g., a higher strength) has been rejected. However, products that were prescribed and dispensed to patients before the marketing authorization was refused and the marketability ended can still be used within their shelf life. 

The TAO does not provide for any extension of the deadlines specified in Section 3 (4) TAO. If product development is not completed within these deadlines, further development will only be possible outside the TAO. This means that at a later date, once product development has been completed, an application for approval of marketing authorization can be submitted in accordance with Sections 21 ff. AMG [14]. Until then, however, the product cannot be further distributed, but may only be used in the context of approved clinical trials. 

## Current status of proceedings 

Based on the results of clinical trials conducted under the transitional provisions of the TAO, two tree pollen AIT products were granted a marketing authorization in Germany in 2018. Currently (as of August 8, 2025), 40 AIT products are still marketable under the transitional provisions of the TAO: 10 products for the treatment of allergies to house dust mites, 10 for tree pollen allergies, 16 for grass pollen allergies, and 4 mixtures containing non-homologous allergen groups (https://www.pei.de/SharedDocs/Downloads/DE/arzneimittel/liste-therapie-allergen-nach-tav-de-en.pdf). For 8 of these products, the pharmaceutical companies have declared withdrawal of their MAAs: 3 products as of October 1, 2025, and 5 products as of January 31, 2026. Withdrawals and refusals of MAA are published on the PEI website in accordance with Section 34 (1b) sentence 1 AMG (https://www.pei.de/EN/newsroom/publications-medicines/34-amg-en/34-amg-content.html). 

The PEI is not informed about all study activities worldwide. In accordance with its areas of responsibility, only those clinical trials that are to be conducted in Germany are submitted to the PEI for approval. Before the end of the granted deadlines for resolving deficiencies and the final comprehensive assessment of the updated or newly submitted marketing authorization dossiers, the PEI is therefore unable to make public statements on whether the remaining 32 products (after the above-mentioned withdrawals of MAA) that are being assessed under the TAO will be granted marketing authorization or will be rejected. 

For AIT products that are not undergoing further clinical development within the product-specific deadlines set by the TAO, but are instead intended to be withdrawn from the market in the near future, early communication by pharmaceutical companies is encouraged in order to inform allergists, pharmacies, and patients in a timely and transparent manner so that treatment decisions regarding new prescriptions, treatment completion, or treatment changes can be made in a well-informed manner. In the event that a treatment change is necessary, authorized AIT products, listed by route of administration (SLIT https://www.pei.de/EN/medicinal-products/allergens/therapy-sublingual/sublingual-therapy-node.html or SCIT https://www.pei.de/EN/medicinal-products/allergens/therapy-subcutaneous/subcutaneous-therapy-node.html) and allergen source, can be found on the PEI website. 

## Outlook 

The final product-specific deadlines for completing marketing authorization dossiers and submitting meaningful clinical data as part of product development under the transitional provision of the TAO will expire in the course of 2026. The PEI will then assess the available data on a product-specific basis and decide whether to grant or decline marketing authorization. All essential information on the marketing authorization decision will be summarized in the public assessment reports (PARs) and will be available to users and all interested parties on the federal and state drug information portal (online platform PharmNet.Bund; https://www.pharmnet-bund.de/). These can be found under the respective medicinal product in the supplementary documents in addition to the publicly available Summary of Product Characteristics (SmPC) and patient information leaflet (PIL). In the event of marketing authorization rejection, the reasons for rejection are published on the PEI website (https://www.pei.de/EN/newsroom/publications-medicines/34-amg-en/34-amg-content.html). Pharmaceutical companies may withdraw their MAA before the assessment has been completed. In this case, the date of withdrawal, but no reasons for the withdrawal, will be published on the PEI website (https://www.pei.de/EN/newsroom/publications-medicines/34-amg-en/34-amg-content.html). 

## Conclusion 

The final stretch of the marketing authorization process under the TAO is a very dynamic phase. For different products under the TAO, a simultaneous occurrence of different processes is to be expected: from completion of clinical development, updating of dossiers, or withdrawal of MAAs by pharmaceutical companies, as well as assessment by the competent authority, followed by granting of new marketing authorizations or refusal of marketing authorization. The legal requirements of the TAO have stimulated a lively study activity, which has contributed significantly to improving the evidence base for AIT products on the market and to a sustainable improvement in the care of allergy patients. 

## Authors’ contributions 

Vera Mahler (VM), Diana Hartenstein (DH), Iris Lauer (IL), Stefan Vieths (SV), Claudia Ruoff (CR), Julia Zimmer (JZ) and Susanne Kaul (SK) contributed in acquisition and interpretation of data for the work. VM drafted the manuscript. DH, IL, SV, CR, JZ, SK and VM were revising it critically for important intellectual content. All authors have given approval for publication of the content and agree to be accountable for all aspects of the work. 

## Funding 

Resources of PEI. No external funding. 

## Conflict of interest 

The authors declare that there is no conflict of interest.
